# Supplementary material for: Hippocampal subfield volumes in abstinent men and women with a history of alcohol use disorder
Source: PLoS One. 2020 Aug 10;15(8):e0236641. doi: 10.1371/journal.pone.0236641 (PMC7416961; doi:10.1371/journal.pone.0236641)
Supplement: S1 Table — Means and standard deviations (SD) are provided for the hippocampal regional volumes of AUDw (N = 31) and AUDm (N = 36) (women and men with a history of Alcohol Use Disorder), along with NCw (N = 30) and NCm (N = 33) (women and men without a history of AUD). Abbreviations: CA1 through 4 = cornu ammonis 1 through 4; DG = dentate gyrus; HATA = hippocampal-amygdaloid transition area. *Indicates regions where AUD < NC, p < 0.0042. (DOCX) [file pone.0236641.s001.docx]

|  | AUDw (N=31) | | AUDm (N=36) | | NCw (N=31) | | NCm (N=33) | |
| --- | --- | --- | --- | --- | --- | --- | --- | --- |
| Hippocampus  Region | Mean | (SD) | Mean | (SD) | Mean | (SD) | Mean | (SD) |
| Hippocampal tail* | 516.49 | (56.95) | 515.27 | (72.36) | 545.05 | (48.79) | 544.78 | (82.81) |
| Subiculum | 416.10 | (46.62) | 407.40 | (41.44) | 430.39 | (29.07) | 428.75 | (33.04) |
| CA1* | 603.45 | (63.31) | 603.18 | (57.16) | 634.30 | (50.37) | 638.04 | (47.46) |
| Hippocampal fissure | 142.29 | (16.03) | 149.72 | (23.63) | 147.16 | (25.44) | 138.19 | (22.97) |
| Presubiculum | 288.95 | (38.88) | 278.32 | (39.30) | 300.50 | (28.28) | 292.49 | (36.36) |
| Parasubiculum | 54.15 | (9.41) | 55.86 | (11.44) | 58.19 | (7.75) | 59.35 | (9.85) |
| Molecular layer* | 547.90 | (55.76) | 539.08 | (55.00) | 573.22 | (40.65) | 574.25 | (45.51) |
| DG | 290.78 | (29.45) | 289.76 | (30.21) | 302.76 | (22.21) | 308.26 | (24.38) |
| CA2+3 | 211.00 | (21.03) | 210.65 | (25.99) | 219.28 | (20.24) | 226.71 | (21.39) |
| CA4 | 251.62 | (24.43) | 250.97 | (24.96) | 261.87 | (18.79) | 266.95 | (19.84) |
| Fimbria | 68.16 | (14.93) | 62.89 | (20.60) | 73.39 | (11.45) | 71.86 | (14.82) |
| HATA | 58.06 | (7.33) | 56.66 | (9.04) | 61.75 | (6.39) | 62.44 | (6.54) |
| Whole hippocampus | 3306.67 | (302.24) | 3270.04 | (319.00) | 3460.70 | (206.19) | 3473.89 | (268.89) |

S1 Table. Regional volumes adjusted for estimated total intracranial volume (eTIV).

Means and standard deviations (SD) are provided for the hippocampal regional volumes of AUDw (N=31) and AUDm (N=36) (women and men with a history of Alcohol Use Disorder), along with NCw (N=30) and NCm (N=33) (women and men without a history of AUD). Abbreviations: CA1 through 4 = cornu ammonis 1 through 4; DG = dentate gyrus; HATA = hippocampal-amygdaloid transition area. *Indicates regions where AUD < NC, *p* < 0.0042.
