# Supplementary material for: Hippocampal subfield volumes in abstinent men and women with a history of alcohol use disorder
Source: PLoS One. 2020 Aug 10;15(8):e0236641. doi: 10.1371/journal.pone.0236641 (PMC7416961; doi:10.1371/journal.pone.0236641)
Supplement: S2 Table — The analysis of variance obtained from the model indicated significant group-by-region-by-age and gender-by-region-by-age interactions for volumes. Colons indicate interaction effects. Abbreviations: Sum Sq = sums of squares; Mean Sq = mean square; NumDF = numerator degrees of freedom; DenDF = denominator degrees of freedom; Pr(>F) = probability > F (i.e., p value). (DOCX) [file pone.0236641.s002.docx]

|  | Sum Sq | Mean Sq | NumDF | DenDF | F value | Pr(>F) |
| --- | --- | --- | --- | --- | --- | --- |
| group | 1772.18 | 1772.18 | 1.00 | 123.00 | 2.44 | 0.12 |
| region | 4207711.27 | 382519.21 | 11.00 | 1364.00 | 526.07 | 0.00 |
| age | 24797.22 | 24797.22 | 1.00 | 123.00 | 34.10 | 0.00 |
| gender | 569.42 | 569.42 | 1.00 | 123.00 | 0.78 | 0.38 |
| group:region | 12467.15 | 1133.38 | 11.00 | 1364.00 | 1.56 | 0.11 |
| group:age | 4740.40 | 4740.40 | 1.00 | 123.00 | 6.52 | 0.01 |
| region:age | 131786.39 | 11980.58 | 11.00 | 1364.00 | 16.48 | 0.00 |
| region:gender | 15165.40 | 1378.67 | 11.00 | 1364.00 | 1.90 | 0.04 |
| age:gender | 321.72 | 321.72 | 1.00 | 123.00 | 0.44 | 0.51 |
| group:gender | 313.61 | 313.61 | 1.00 | 123.00 | 0.43 | 0.51 |
| group:region:age | 21861.75 | 1987.43 | 11.00 | 1364.00 | 2.73 | 0.00 |
| region:age:gender | 13163.70 | 1196.70 | 11.00 | 1364.00 | 1.65 | 0.08 |
| group:region:gender | 5718.29 | 519.84 | 11.00 | 1364.00 | 0.71 | 0.73 |
| group:age:gender | 529.23 | 529.23 | 1.00 | 123.00 | 0.73 | 0.40 |

S2 Table. Analysis of variance for the primary model of our study.

The analysis of variance obtained from the model indicated significant group-by-region-by-age and gender-by-region-by-age interactions for volumes. Colons indicate interaction effects. Abbreviations: Sum Sq = sums of squares; Mean Sq = mean square; NumDF = numerator degrees of freedom; DenDF = denominator degrees of freedom; Pr(>F) = probability > F (i.e., *p* value).
