# Supplementary material for: Hippocampal subfield volumes in abstinent men and women with a history of alcohol use disorder
Source: PLoS One. 2020 Aug 10;15(8):e0236641. doi: 10.1371/journal.pone.0236641 (PMC7416961; doi:10.1371/journal.pone.0236641)
Supplement: S3 Table — The analysis of variance obtained from the model indicated a significant group-by-region-by-IMI interaction for volumes. Colons indicate interaction effects. Abbreviations: Sum Sq = sums of squares; Mean Sq = mean square; NumDF = numerator degrees of freedom; DenDF = denominator degrees of freedom; Pr(>F) = probability > F (i.e., p value); IMI = Wechsler Memory Scale Immediate Memory Index. (DOCX) [file pone.0236641.s003.docx]

|  | Sum Sq | Mean Sq | NumDF | DenDF | F value | Pr(>F) |
| --- | --- | --- | --- | --- | --- | --- |
| group | 666.63 | 666.63 | 1.00 | 119.00 | 0.93 | 0.34 |
| region | 1080255.08 | 98205.01 | 11.00 | 1331.00 | 136.49 | 0.00 |
| IMI | 518.52 | 518.52 | 1.00 | 119.00 | 0.72 | 0.40 |
| gender | 1204.93 | 1204.93 | 1.00 | 119.00 | 1.67 | 0.20 |
| age | 20154.11 | 20154.11 | 1.00 | 119.00 | 28.01 | 0.00 |
| group:region | 16483.29 | 1498.48 | 11.00 | 1331.00 | 2.08 | 0.02 |
| group:IMI | 209.23 | 209.23 | 1.00 | 119.00 | 0.29 | 0.59 |
| region:IMI | 2826.71 | 256.97 | 11.00 | 1331.00 | 0.36 | 0.97 |
| region:gender | 16835.19 | 1530.47 | 11.00 | 1331.00 | 2.13 | 0.02 |
| gender:age | 904.51 | 904.51 | 1.00 | 119.00 | 1.26 | 0.26 |
| region:age | 99239.83 | 9021.80 | 11.00 | 1331.00 | 12.54 | 0.00 |
| group:gender | 6.58 | 6.58 | 1.00 | 119.00 | 0.01 | 0.92 |
| group:age | 2158.10 | 2158.10 | 1.00 | 119.00 | 3.00 | 0.09 |
| group:region:IMI | 19321.94 | 1756.54 | 11.00 | 1331.00 | 2.44 | 0.01 |
| region:gender:age | 14811.29 | 1346.48 | 11.00 | 1331.00 | 1.87 | 0.04 |
| group:gender:age | 25.41 | 25.41 | 1.00 | 119.00 | 0.04 | 0.85 |
| group:region:gender | 2783.85 | 253.08 | 11.00 | 1331.00 | 0.35 | 0.97 |

S3 Table. Analysis of variance for a secondary model of our study, which includes the Immediate Memory Index.

The analysis of variance obtained from the model indicated a significant group-by-region-by-IMI interaction for volumes. Colons indicate interaction effects. Abbreviations: Sum Sq = sums of squares; Mean Sq = mean square; NumDF = numerator degrees of freedom; DenDF = denominator degrees of freedom; Pr(>F) = probability > F (i.e., *p* value); IMI = Wechsler Memory Scale Immediate Memory Index.
