# Supplementary material for: Hippocampal subfield volumes in abstinent men and women with a history of alcohol use disorder
Source: PLoS One. 2020 Aug 10;15(8):e0236641. doi: 10.1371/journal.pone.0236641 (PMC7416961; doi:10.1371/journal.pone.0236641)
Supplement: S4 Table — The analysis of variance obtained from the model indicated a significant group-by-region-by-DMI interaction for volumes. Colons indicate interaction effects. Abbreviations: Sum Sq = sums of squares; Mean Sq = mean square; NumDF = numerator degrees of freedom; DenDF = denominator degrees of freedom; Pr(>F) = probability > F (i.e., p value); DMI = Wechsler Memory Scale Delayed Memory Index. (DOCX) [file pone.0236641.s004.docx]

|  | Sum Sq | Mean Sq | NumDF | DenDF | F value | Pr(>F) |
| --- | --- | --- | --- | --- | --- | --- |
| group | 1383.87 | 1383.87 | 1.00 | 119.00 | 1.94 | 0.17 |
| region | 1009812.04 | 91801.09 | 11.00 | 1331.00 | 128.79 | 0.00 |
| DMI | 687.47 | 687.47 | 1.00 | 119.00 | 0.96 | 0.33 |
| gender | 1363.08 | 1363.08 | 1.00 | 119.00 | 1.91 | 0.17 |
| age | 20672.24 | 20672.24 | 1.00 | 119.00 | 29.00 | 0.00 |
| group:region | 24362.93 | 2214.81 | 11.00 | 1331.00 | 3.11 | 0.00 |
| group:DMI | 831.53 | 831.53 | 1.00 | 119.00 | 1.17 | 0.28 |
| region:DMI | 2945.52 | 267.77 | 11.00 | 1331.00 | 0.38 | 0.97 |
| region:gender | 21011.18 | 1910.11 | 11.00 | 1331.00 | 2.68 | 0.00 |
| gender:age | 1026.61 | 1026.61 | 1.00 | 119.00 | 1.44 | 0.23 |
| region:age | 102699.22 | 9336.29 | 11.00 | 1331.00 | 13.10 | 0.00 |
| group:gender | 32.61 | 32.61 | 1.00 | 119.00 | 0.05 | 0.83 |
| group:age | 1920.62 | 1920.62 | 1.00 | 119.00 | 2.69 | 0.10 |
| group:region:DMI | 28614.52 | 2601.32 | 11.00 | 1331.00 | 3.65 | 0.00 |
| region:gender:age | 18580.66 | 1689.15 | 11.00 | 1331.00 | 2.37 | 0.01 |
| group:gender:age | 67.42 | 67.42 | 1.00 | 119.00 | 0.09 | 0.76 |
| group:region:gender | 2829.17 | 257.20 | 11.00 | 1331.00 | 0.36 | 0.97 |

S4 Table. Analysis of variance for a secondary model of our study, which includes the Delayed Memory Index.

The analysis of variance obtained from the model indicated a significant group-by-region-by-DMI interaction for volumes. Colons indicate interaction effects. Abbreviations: Sum Sq = sums of squares; Mean Sq = mean square; NumDF = numerator degrees of freedom; DenDF = denominator degrees of freedom; Pr(>F) = probability > F (i.e., *p* value); DMI = Wechsler Memory Scale Delayed Memory Index
