# Supplementary material for: Hippocampal subfield volumes in abstinent men and women with a history of alcohol use disorder
Source: PLoS One. 2020 Aug 10;15(8):e0236641. doi: 10.1371/journal.pone.0236641 (PMC7416961; doi:10.1371/journal.pone.0236641)
Supplement: S5 Table — The analysis of variance obtained from the model indicated a significant gender-by-region-by-LOS interaction for volumes, for the AUD group. Colons indicate interaction effects. Abbreviations: Sum Sq = sums of squares; Mean Sq = mean square; NumDF = numerator degrees of freedom; DenDF = denominator degrees of freedom; Pr(>F) = probability > F (i.e., p value;.DHD = duration of heavy drinking; DD = daily drinks; LOS = length of sobriety. (DOCX) [file pone.0236641.s005.docx]

|  | Sum Sq | Mean Sq | NumDF | DenDF | F value | Pr(>F) |
| --- | --- | --- | --- | --- | --- | --- |
| gender | 268.85 | 268.85 | 1.00 | 57.00 | 0.37 | 0.55 |
| region | 1038965.99 | 94451.45 | 11.00 | 627.00 | 129.73 | 0.00 |
| DHD | 68.26 | 68.26 | 1.00 | 57.00 | 0.09 | 0.76 |
| DD | 892.90 | 892.90 | 1.00 | 57.00 | 1.23 | 0.27 |
| LOS | 85.70 | 85.70 | 1.00 | 57.00 | 0.12 | 0.73 |
| age | 6464.69 | 6464.69 | 1.00 | 57.00 | 8.88 | 0.00 |
| gender:region | 7364.98 | 669.54 | 11.00 | 627.00 | 0.92 | 0.52 |
| gender:DHD | 200.65 | 200.65 | 1.00 | 57.00 | 0.28 | 0.60 |
| region:DHD | 4368.67 | 397.15 | 11.00 | 627.00 | 0.55 | 0.87 |
| gender:DD | 15.44 | 15.44 | 1.00 | 57.00 | 0.02 | 0.88 |
| region:DD | 8473.59 | 770.33 | 11.00 | 627.00 | 1.06 | 0.39 |
| gender:LOS | 2351.24 | 2351.24 | 1.00 | 57.00 | 3.23 | 0.08 |
| region:LOS | 17863.58 | 1623.96 | 11.00 | 627.00 | 2.23 | 0.01 |
| gender:age | 968.00 | 968.00 | 1.00 | 57.00 | 1.33 | 0.25 |
| region:age | 35241.79 | 3203.80 | 11.00 | 627.00 | 4.40 | 0.00 |
| gender:region:DHD | 3326.68 | 302.43 | 11.00 | 627.00 | 0.42 | 0.95 |
| gender:region:DD | 6316.31 | 574.21 | 11.00 | 627.00 | 0.79 | 0.65 |
| gender:region:LOS | 18721.68 | 1701.97 | 11.00 | 627.00 | 2.34 | 0.01 |
| gender:region:age | 10323.07 | 938.46 | 11.00 | 627.00 | 1.29 | 0.23 |

S5 Table. Analysis of variance for a secondary model of our study, which includes the AUD groups’ drinking history (DHD, DD, and LOS).

The analysis of variance obtained from the model indicated a significant gender-by-region-by-LOS interaction for volumes, for the AUD group. Colons indicate interaction effects. Abbreviations: Sum Sq = sums of squares; Mean Sq = mean square; NumDF = numerator degrees of freedom; DenDF = denominator degrees of freedom; Pr(>F) = probability > F (i.e., *p* value;.DHD = duration of heavy drinking; DD = daily drinks; LOS = length of sobriety .
