# Supplementary material for: Hippocampal subfield volumes in abstinent men and women with a history of alcohol use disorder
Source: PLoS One. 2020 Aug 10;15(8):e0236641. doi: 10.1371/journal.pone.0236641 (PMC7416961; doi:10.1371/journal.pone.0236641)
Supplement: S6 Table — Counts of participants are given for each level of the measures listed for AUDw (N = 31) and AUDm (N = 36) (women and men with a history of Alcohol Use Disorder), along with NCw (N = 31) and NCm (N = 33) (women and men without a history of AUD). Confounded and unconfounded subsample assignment is described in the Methods. The five participants listed with Five Year Drug History of ‘once per week or more’ were occasional marijuana users. First Degree History was indicated by participant endorsement of ‘Alcoholic’ for mother, father, sibling, or children. Second Degree History was indicated by participant endorsement of ‘Alcoholic’ for grandparents, aunts, uncles, or grandchildren. (DOCX) [file pone.0236641.s006.docx]

| Measure | Level | AUDm | AUDw | NCm | NCw |
| --- | --- | --- | --- | --- | --- |
| Confounding | Confounded | 22 | 19 | 6 | 6 |
| Confounding | Unconfounded | 14 | 12 | 27 | 25 |
| Current Smoking | No | 22 | 19 | 29 | 29 |
| Current Smoking | Yes | 14 | 12 | 4 | 2 |
| Five Year Drug History | No | 31 | 27 | 31 | 28 |
| Five Year Drug History | Less than once per week | 4 | 2 | 1 | 2 |
| Five Year Drug History | Once per week or more | 1 | 2 | 1 | 1 |
| Lifetime Drug History | No | 9 | 11 | 26 | 24 |
| Lifetime Drug History | Less than once per week | 11 | 7 | 3 | 3 |
| Lifetime Drug History | Once per week or more | 16 | 13 | 4 | 4 |
| Mother History | Alcoholic | 8 | 9 | 2 | 6 |
| Mother History | Alcoholic and drug abuse | 2 | 3 | 0 | 2 |
| Mother History | Drug abuse | 0 | 1 | 0 | 0 |
| Mother History | Never | 6 | 5 | 7 | 8 |
| Mother History | Social drinker | 20 | 13 | 24 | 15 |
| Father History | Alcoholic | 16 | 19 | 7 | 7 |
| Father History | Alcoholic and drug abuse | 3 | 3 | 0 | 2 |
| Father History | Don't know | 3 | 1 | 2 | 3 |
| Father History | Never | 1 | 2 | 3 | 4 |
| Father History | Social drinker | 13 | 6 | 21 | 15 |
| First Degree History | No | 11 | 2 | 25 | 16 |
| First Degree History | Yes | 25 | 29 | 8 | 15 |
| Second Degree History | No | 14 | 5 | 22 | 19 |
| Second Degree History | Yes | 22 | 26 | 11 | 12 |

S6 Table. Additional participant characteristics.

Counts of participants are given for each level of the measures listed for AUDw (N=31) and AUDm (N=36) (women and men with a history of Alcohol Use Disorder), along with NCw (N=31) and NCm (N=33) (women and men without a history of AUD). Confounded and unconfounded subsample assignment is described in the Methods. The five participants listed with Five Year Drug History of ‘once per week or more’ were occasional marijuana users. First Degree History was indicated by participant endorsement of ‘Alcoholic’ for mother, father, sibling, or children. Second Degree History was indicated by participant endorsement of ‘Alcoholic’ for grandparents, aunts, uncles, or grandchildren.
