# Supplementary material for: Hippocampal subfield volumes in abstinent men and women with a history of alcohol use disorder
Source: PLoS One. 2020 Aug 10;15(8):e0236641. doi: 10.1371/journal.pone.0236641 (PMC7416961; doi:10.1371/journal.pone.0236641)
Supplement: Attachment — Submitted filename: PONE-D-19-32308R1-reply.docx [file pone.0236641.s009.docx]

**Kayle S. Sawyer, Ph.D.**

**Boston University School of Medicine, L-1004**

**72 East Concord Street**

**Boston, MA 02118**

May 28, 2020

Stephen D. Ginsberg, Ph.D., Section Editor, PLOS ONE

Nathan S. Kline Institute for Psychiatric Research

New York, NY

Dear Dr. Ginsberg:

We thank the editor and the reviewers for their valuable comments regarding our manuscript entitled “Hippocampal Subfield Volumes in Abstinent Men and Women with a History of Alcohol Use Disorder” (PONE-D-19-32308). We also thank the editor and the reviewers for the effort and time put into the review of the manuscript. We have addressed each comment below and accordingly revised the manuscript. We believe that the changes made in this revision have created a more detailed theoretical rationale for our investigation, improved the quality of our manuscript, and strengthened our conclusions.

REVIEWER 1

1. **Reviewer 1: The manuscript describes an analysis of the hippocampal subfields volumes in a large sample of AUD patients compared with controls. The authors also examined the relationships with general memory scores and alcohol history. The manuscript is well-written and easy to follow despite the numerous analyses. I particularly appreciate the strategy for the statistical analyses: large samples representative of the clinical population and smaller ones much more carefully selected to avoid the effects of confounding factors. The statistical analyses are rigorously chosen. The results are sometimes surprising and counterintuitive but the authors manage to discuss them quite well.**

Authors: We appreciate the complimentary assessment.

2. **Reviewer 1: There is no page number, neither line number, which makes it difficult to refer to.**

Authors: We have included page numbers and line numbers.

*Introduction:*

3. **Reviewer 1: “negative association between… (clinical vs subclinical)”: could you detail?**

Authors: In the first paragraph of the Introduction, and based upon the information provided in the meta-analysis we cited therein, we changed the wording to distinguish clinical and subclinical use as follows: “...and a meta-analysis [[1]](https://paperpile.com/c/58tliY/i698P) found a negative association between total hippocampal volume and degree of alcohol use (i.e., reduced hippocampal volume in individuals with a clinical diagnosis of AUD, but not in individuals with problematic use such as binge drinking).

4. **Reviewer 1: “This processing stream is repeated… ventral to dorsal”: could you specify? Which processing stream? What is organized from anterior to posterior? Do you refer to the nature of the stimuli that are processed? Or to the nature of the process per se?**

Authors: The wording we used was unclear, and we modified it. The “processing stream that is repeated” was intended to refer to the entire circuitry shown in Figure 1B, encompassing both the direct and the trisynaptic pathways. When we referred to “anterior to posterior organization,” we intended that the reader think about the circuitry shown in this slice being repeated over and over, from the anterior to the posterior extents of the hippocampus. We meant to refer to the nature of “the process *per se*,” not some specific stimuli. That is, the circuit diagram shown in the Figure 1B slice would look similar if it were taken from the anterior portion or the posterior portion of the hippocampus. We removed the confusing term “axis,” and added new text to the fourth paragraph of the Introduction, as follows:

“Both the direct and the trisynaptic pathways exist in parallel throughout the anterior, middle, and posterior hippocampus. That is, the slice shown in Figure 1B would look similar were the slice taken from a more anterior or from a more posterior section through the hippocampus.”

We also simplified part of the third paragraph, as follows:

“The anterior portion (analogous to *ventral* in the rodent) has been considered to be involved in a wide variety of contexts including stress, emotion, and affect [42], while the posterior portion (analogous to *dorsal* in the rodent) has been demonstrated to have involvement in spatial processing (Nadel et al., 2013).”

5. **Reviewer 1: “the problems contributing to, and resulting from, AUD”: the fact that altered memory abilities result from AUD makes sense. Can you explain how shrinkage of specific hippocampal subfield and altered memory abilities contribute to AUD? You explain this point only in the discussion.**

Authors: Because this was ambiguous, we changed the text in the seventh paragraph of the Introduction as follows:

“In summary, because the evidence regarding AUD-related hippocampal subfield dysmorphology is inconclusive, we sought to clarify the volumetric and functional abnormalities, and their associations with age, gender, and memory. Ultimately, we hope that the findings will help lead to an increased understanding of how subregional hippocampal shrinkage contributes to altered memory abilities in AUD men and women as they age.”

*Methods:*

6. **Reviewer 1: Why did the inclusion criteria require a minimum of 4 weeks of abstinence? The withdrawal syndrome is over well before 4 weeks.**

Authors: In the fourth paragraph of the Methods/Participants section of the manuscript, we now explain the minimum duration of four weeks of abstinence as follows: “...a minimum of four weeks of abstinence prior to testing, which is important for obtaining stable levels of performance after ethanol and its metabolites have been eliminated from the body [[2]](https://paperpile.com/c/58tliY/p5gho).”

7. **Reviewer 1: Why did the authors focus on these two memory indexes? What about the three other ones? Do they have specific hypotheses with these ones?**

Authors: We appreciate the concern about the general nature of these indices. We did not have specific hypotheses regarding the additional indices. However, researchers are welcome to explore additional relationships with the data and code we provided. We now include this point in the Limitations, as follows:

“We also limited the scope of this study with regard to the number of cognitive assessments we considered in our analyses, i.e., Full Scale IQ (Wechsler, 1997) and two inclusive measures of memory, i.e., immediate and delayed memory (Wechsler, 1997), that we hypothesized to be positively associated with volumetric measures. Other WAIS and WMS measures can be found in the available data and code: <https://gitlab.com/kslays/moblab-hippocampus>.”

8. **Reviewer 1: What are the “composite scores” of the WAIS? Not mentioned in Table 1 (just full IQ).**

Authors: In the final section of the Methods, we clarified the wording. We deleted the words “Composite Scores were analyzed...” in favor of the following: “IMI and DMI scores were analyzed...”, so the legend of Table 1 is now consistent with the text. We did not examine composite scores of the WAIS, and we list only the Full Scale IQ as one of the participant characteristics.

9. **Reviewer 1: Since the authors explain that the different hippocampal subfields have different cognitive functions, why did they use a very global memory scale, which does not permit to evaluate these specific cognitive/memory processes?**

Authors: Because this comment is similar to Item #5 above, we combined our revision into a single reply that is added to the Limitations.

*Discussion:*

10. **Reviewer 1: Previous studies reporting shrinkage of other hippocampal regions included patients with shorter LOS. I do not think that with an average of 7 years of sobriety, results can reflect acute effects of alcohol.**

Authors: We clarified the wording of the Discussion (*Influence of Participant Characteristics*) to the following: “However, abstinence durations for the AUD participants examined in those papers generally were considerably shorter than the average abstinence durations of the participants in the present study (7.1 years). Therefore, results reported in the earlier studies could reflect effects associated with early sobriety.”

*Tables/Figures*:

11. **Reviewer 1: Table 1 is difficult to read because of too many abbreviations. Please, include the statistics in the table to facilitate the reading.**

Authors: We are sorry that Table 1 was unclear. We changed the abbreviations of WAIS FSIQ to “Full Scale IQ” and WMS IMI and DMI to “Immediate Memory” and “Delayed Memory”; and EtOH to “ethanol.” Additionally, we have added superscripts to indicate significant differences.

12. **Reviewer 1: LOS is supposed to be in years given the legend of Table 1. Have the women AUD really been sober in average for 11 years (with a maximum of 36 years)? If so, the sample (even in men AUD) is very heterogeneous regarding the LOS, which is nice to evaluate recovery effect but makes it difficult to state on the presence/absence of alterations at a specific time point. For example, the 5% smaller volumes of different hippocampal fields corresponds to the volumes at 4 weeks (certainly not), 6 months, 6 years of sobriety?**

Authors: Indeed, the AUD women have been sober for an average of 11 years, with a range of 0.1 to 36.0 years. In Table 1, the LOS is labeled in years, and the volume differences reflect an average LOS of 7.1 years. We added the following sentence to the description of the Participant Characteristics section in the Results: “The average duration of the most recent period of sobriety for AUD participants was 7.1 years.” Also, in the Limitations, we added: “Relatedly, our sample had a heterogeneous LOS, which prevented us from specifying time points corresponding to the percentage of inferred changes we reported in subfield volumes.”

13. **Reviewer 1: I cannot find the * in Figure 2 (as indicated in the legend).**

Authors: We apologize for this error. We had created the image in R and added the asterisk (*) using Inkscape, but accidentally uploaded the original image instead of the image with the asterisk included.

14. **Reviewer 1: Figure 3: B appears before A in the text.**

Authors: We modified Figure 3 so that the hippocampal tail is A, and CA1 is B, in concordance with the order in which they are referenced in the text.

REVIEWER 2

1. **Reviewer 2: This paper looks at hippocampal subfield volumes in AUD and tries to elucidate age-sex-group relationships. This is a fairly new area with few prior work, aided by the recent availability of hippocampal subfield segmentation software (Freesurfer).** **There are two major limitations of the paper which could potentially have contributed to their results being different from prev. work and calling into question some of the gender effects claimed here- 1. The MPRAGE T1 have poor slice resolution (1.5mm) but more importantly only T1 data was used for segmentation. Freesurfer 6.0 has been shown to be improved over 5.x only because of the ability to jointly analyze T1 and T2 data. This is clearly the motivation of Iglesias et al. when they developed FS6.0. However, in this work only T1 was used which puts into question the validity of volumes measures of small structures like the molecular layer which are hardly even visualized in T1. It is likely this cannot be corrected but if the authors have access to the T2 data, they should use it in the FS pipeline to significantly improve accuracy. Otherwise, they cannot claim the superior segmentation of 6.0 over 5.X.**

Authors: T2 scans were not available for this dataset. However, in concordance with the reviewer’s suggestion, we added the following sentence to the Limitations as follows: “Further, the accuracy of our automated segmentations was limited, because T2 scans were unavailable. The segmentation procedure we used (developed by Iglesias et al. (2015)) was built to optionally take advantage of T2 scans. However, Iglesias and colleagues had built the segmentation classifier used in the FS 6.0 subroutine with T1-only segmentation in mind (see page 133 of their paper).” We also added the following sentence to the Methods: “T2 scans were unavailable for use in improving segmentation accuracy beyond the accuracy obtained with the T1 scans.”

We wish to note that Iglesias and colleagues had built the segmentation classifier used in the FS 6.0 subroutine with T1-only segmentation in mind, as described in their paper as follows: “The use of a simple, linear classifier such as LDA ensures that the classification accuracy is mainly determined by the quality of the input data (i.e., the subregion volumes) rather than stochastic variations in the classifier.”

2. **Reviewer 2: The other major and perhaps critical limitation (which can be fixed !) is the correction for ICV. It has been shown in several papers (Pintzka et al. Front Neurosci. 2015; 9: 238. Voevodskaya Front. Aging Neurosci., 07 October 2014 ) that the method of ICV correction is critical esp. in determining sex correlations to volumes. It has been shown either the residual or the ANCOVA method are the most accurate and the proportions based method (i.e. vol/ICV) the least accurate and leading to confounding results. Since one of the main claims of this paper is sex specific effects, this has to be addressed or the results are questionable. The residual method is very easy to implement (fit the control volumes to the ICVs, use the slope s to correct AUD vols as V' = V - s*(ICV- mean ICV).**

Authors: We thank Reviewer 2 for this insight on ICV correction, and we have implemented the residual method for ICV correction using the procedure suggested (using our measure of estimated total intracranial volume, eTIV). With the new method, data for one of the two participants that had been excluded as an outlier (> 4 SD) were no longer outlier data, so the sample size increased by 1. Using the new values, we changed all of the results presented in the manuscript. The new data and code are provided at https://gitlab.com/kslays/moblab-hippocampus, with separate files containing the code for the new main results using the residual method, the code for the matched sample using the residual method, the code for the unconfounded sample with the residual method, and the code for the old proportion method. The results obtained after using the new ICV correction procedure were similar to those obtained previously using ICV proportions.

*Introduction:*

3. **Reviewer 2: It is possible that a correct and accurate ICV correction will eliminate the gender effect and possibly the reason why there were no prev. gender specific effects reported in prev. studies. [See major comment above].**

Authors: We applied the residual method of ICV correction, as suggested by Reviewer 2, and then found no gender-by-region-by-age interaction, nor any gender effects for individual subfields after correction for multiple comparisons. We have updated all the results in the text, and therefore, removed the gender specific results.

4. **Reviewer 2: The introduction is a bit too long and rambling and didactic. Anatomy of the hippocampal subfields etc can be removed and summarized in the Discussion.**

Authors: We would like to mention that Reviewer 3 wrote, **“the rationale is clearly laid out in the Introduction, relevant literature and background is reviewed.”** In considering the suggestions of both reviewers, we believe that our presentation in the Introduction and Discussion are valuable, and should be included to help readers understand and interpret our results. We made our decision in the context of a limited literature on hippocampal morphometrics in AUD research, especially literature accompanied by concurrent information about hippocampal subfield function.

*Abstract*:

5. **Reviewer 2: "higher scores" please clarify what these scores are**

Authors: We apologize for the confusion. We now clarify our statement in the Abstract as follows: “Higher scores on tests of immediate and delayed memory were associated with smaller volumes in the AUD group, but larger volumes in the NC group.”

*Results:*

6. **Reviewer 2: "AUD group had 5.18%, 5.08%..." -- if there are sex effects, shouldn't this be separated by sex? Similarly in the next para, it says Women had 8.43%, 4.99% etc but this should also be separated by group to see what those effects are (if there are) and then combined if there are indeed no effects.**

Authors: Following the new ICV correction, although the gender-by-region interaction was still significant (*p* = 0.04), we did not find sex effects for any particular subfields (all *p* > 0.0042). Also, as now described in the updated manuscript, no group-by-gender interactions were significant. Thus, we only discuss group-by-region-by-age interactions.

7. **Reviewer 2: For the age effects, it suddenly switches to %/year. It is not clear how this was calculated. Was it just based on the age spread? I assume there were no multiple time point scans. So this is a bit confusing why units changed from % to % per year.**

Authors: The group differences are reported as percentages in the text to provide a more intuitive measure and to supplement the values provided in the tables. To clarify, we added the following text to the Methods. “For group comparisons, each mean difference in subfield volume was divided by the mean subfield volume for the entire sample. For continuous relationships, each slope was divided by the mean subfield volume for the entire sample.”

8. **Reviewer 2: Again, the accuracy of women having 0.4%/year reduction in CA1 volumes vs. men is questionable based on the way the ICV normaization was done. Same for most other results involving sex differences.**

Authors: Since no sex effects for specific subfields were identified after the new method of ICV normalization, we removed our statement regarding women having 0.4%/year reduction in CA1 volumes vs. men. We recalculated every value reported in the manuscript and removed or updated all of the results.

REVIEWER 3

1. **Reviewer 3: This manuscript examines volumes of hippocampal subfields in a sample of men and women with and without a history of alcohol use disorders. Analyses find several group differences in hippocampal subfield volumes, with main effects of group moderated by interactions with gender, age, and drinking phenotypes. The manuscript is very well written, the rationale is clearly laid out in the Introduction, relevant literature and background is reviewed, and the Discussion is well structured. However, I had some trouble following along with the analyses and results, particularly the multiple 3-way (and even a few 4-way) interactions. I make a few suggestions for this point and have a few additional points below that I hope might improve what is already a strong manuscript.**

Authors: We appreciate and agree with the Reviewer’s summary. In response, we updated the statistical descriptions in response to the individual suggestions (Items 4, 5 and 6), and we also addressed each of the other points below.

2. **Reviewer 3: Please indicate in the Abstract and description of the sample how long participants in the alcohol use disorder group have been abstinent. Length of sobriety is noted in the text/tables—is this the most recent period of sobriety, or the longest length of sobriety in the lifetime?**

Authors: We now clarify in the Abstract and in the description of the sample (see the first paragraph of the Results, *Participant Characteristics*) the following: “The average duration of the most recent period of sobriety for AUD participants was 7.1 years.”

3. **Reviewer 3: Regarding abstinence, how should these results in this sample be interpreted in light of evidence from human and animal studies of hippocampal volume “recovery” following alcohol abstinence? Could it be that some effects of alcohol on the hippocampus are no longer evident in this abstinent sample?**

Authors: In our search of the literature, we were unable to find studies directly focusing on hippocampal subfields volumes in “abstinent” adult nonhuman animals following prolonged ethanol exposures. Regarding human studies, we have added the following text in the first paragraph of the section of the Discussion, *Influence of Participant Characteristics*: “Moreover, those studies of hippocampal subfield volumes [[3–5]](https://paperpile.com/c/58tliY/wibQ+DUun+Upoy) did not examine the *relationship* of long durations of abstinence to volumes. In the few studies that examined total hippocampal volume ‘recovery’, the average length of abstinence was short, varying from a few weeks [[6,7]](https://paperpile.com/c/58tliY/ZkR1+yEmp) to approximately two years [[8]](https://paperpile.com/c/58tliY/jnpw). One study examined a sample of AUD individuals with a history of comorbid psychiatric illnesses and an average abstinence of six years, and the authors found smaller total hippocampal volumes compared to NC individuals [[9]](https://paperpile.com/c/58tliY/DsXu). However, the authors reported only minimal (nonsignificant) differences in hippocampal volumes for abstinent AUD participants without the psychiatric comorbidities. In our sample of AUD participants (many of whom had psychiatric histories as shown in Table S6), it could be that other effects of alcohol consumption were no longer evident after such long periods of abstinence. For the abnormalities in regions found to be in agreement across previous studies and ours (CA1, molecular layer, and subiculum), the combined results support the view that they are vulnerable to chronic long-term AUD.”

4. **Reviewer 3: I had some trouble following the analyses and results. Were the two 3-way interactions of group x gender x region and group x gender x age both included in a single model, as suggested by Table S2? Why was group x gender x memory (or group x age x memory?) not examined, but instead group x region x memory was, as in Tables S3 and S4?**

Authors: Based upon a procedure for the inclusion of interaction terms that was recommended by Dr. Howard Cabral, director of the Biostatistics and Research Design Program of the Boston University Clinical and Translational Sciences Institute, each of the two 3-way interactions (group x gender x region and group x gender x age) were included in a single model, as shown in Table S2. We clarify which effects were in which models by expanding the Methods with the following text: “Four models were examined for this project: (1) The primary model that included the factors of group, gender, age, and subfield; secondary models additionally included (2) immediate memory scores, (3) delayed memory scores, and (4) drinking history measures.” Regarding the memory interactions, we did not have *a-priori* hypotheses regarding group x gender x memory, or regarding group x age x memory. However, as a result of this suggestion by Reviewer 3, we did examine additional models with these interaction terms included. Neither of the interactions was significant for IMI nor for DMI.

5. **Reviewer 3: Related to the above point, I had some trouble following the results for the different 3-way interactions. It seemed that results for main effects were reported (e.g., the third paragraph in the Hippocampal Volumes, Group, Gender, and Age section describes group-level mean differences, but shouldn’t this paragraph be unpacking the 3-way group x region x age model described in the previous paragraph?**

Authors: We appreciate the attention drawn to the 3-way interaction of group x region x age. We apologize for the confusion. In the Methods, we clarified the analyses we conducted by adding the following sentence. “Four statistical models were used for this project: (1) The primary model included the factors of group, age, gender, and subfield; secondary models additionally included (2) immediate memory scores, (3) delayed memory scores, and (4) drinking history measures. For each of the four models, we report findings from the ANOVA, followed by the results from the post hoc analyses (see Results).”
We now use the term “post hoc” as needed in the Methods and Results sections to indicate where we unpack the interactions. For the primary model, we unpack the 3-way group x region x age model in the by comparing the age slopes of the AUD vs NC groups within each of the three significant (*p* < 0.0042) subfields (subiculum, molecular layer, and hippocampal tail). We thought it was important to present post hoc group differences in mean volumes (which were calculated using estimated marginal means within the model) before presenting the group differences in age slopes.
To clarify the order in which we presented the results, we added the following sentence to the beginning of the Results section *Hippocampus Volumes, Group, Age, and Gender*: “Here we report the results for the whole hippocampus, the subfield main model ANOVA, estimated marginal mean differences, comparisons of age slopes, and then for gender effects.” We also revised the wording of the paragraph addressing age slopes to clarify the results for the beginning with the sentence, “The impact of age on subfield volumes interacted significantly with group (the group-by-region-by-age interaction).”

6. **Reviewer 3: I appreciate the efforts to minimize false positive findings, but it wasn’t clear to me why the Bonferroni correction was set as .050/12 (the number of hippocampal subfields) for some analyses but not others. Far more than 12 analyses were conducted, and 12 seems like a fairly arbitrary number. I’m not necessarily suggesting a more stringent p value, but perhaps some additional rationale would be helpful here.**

Authors: Because we had independent a priori hypotheses for each of these four models, we used a *p*-value threshold of 0.05 for the ANOVA results. As described in the preceding item (#5), we now specify these models in the Methods. We did not have individually specified hypotheses for the subfields examined with post hoc analyses, so we corrected these analyses for the multiple comparisons.

That is, to minimize false positive findings, we used a Bonferroni corrected *p* value threshold of 0.05/12 = 0.0042 in the post hoc analyses. After these primary analyses were completed, in order to reduce the number of subsequent tests (and to thereby reduce false positives), we had limited our examination of post hoc analyses for the three memory and drinking history models, to focus *only on the four significant regions identified in the primary model*, i.e., the subiculum, CA1, molecular layer, and hippocampal tail), and therefore, the subsequent post hoc analyses focused on those four subfields. We revised the wording in the Methods to provide this rationale: “In order to reduce the number of subsequent tests (thereby reducing false positives) we limited our examination of post hoc analyses for the three secondary models (involving two memory measures and drinking history) to the subfields found to show significant group differences in the primary model.”

In summary, and reported in the Results, we examined 12 regions post hoc for the primary model, four of which were found to be significant. For subsequent analyses of these four regions, the *p*-values of all significant results were below 0.01, which is lower than the threshold suggested by Bonferroni correction for four regions (0.05/4 = 0.0125). These four regions were examined in the IMI, DMI, and drinking history models, resulting in a total of 12 more examinations of these regions (4 x 3 = 12). So, the first 12 regions were examined with an exploratory analysis threshold of *p* < 0.0042, and the four significant regions were examined three more times, for a total of 24 post hoc examinations of these subfields.

**References**

1.	[Wilson S, Bair JL, Thomas KM, Iacono WG. Problematic alcohol use and reduced hippocampal volume: a meta-analytic review. Psychol Med. 2017;47: 2288–2301.](http://paperpile.com/b/58tliY/i698P)

2.	[Oscar-Berman M, Marinković K. Alcohol: effects on neurobehavioral functions and the brain. Neuropsychol Rev. 2007;17: 239–257.](http://paperpile.com/b/58tliY/p5gho)

3.	[Lee J, Im S-J, Lee S-G, Stadlin A, Son J-W, Shin C-J, et al. Volume of hippocampal subfields in patients with alcohol dependence. Psychiatry Res Neuroimaging. 2016;258: 16–22.](http://paperpile.com/b/58tliY/wibQ)

4.	[Zahr NM, Pohl KM, Saranathan M, Sullivan EV, Pfefferbaum A. Hippocampal subfield CA2+ 3 exhibits accelerated aging in alcohol use disorder: A preliminary study. NeuroImage: Clinical. 2019; 101764.](http://paperpile.com/b/58tliY/DUun)

5.	[Kühn S, Charlet K, Schubert F, Kiefer F. Plasticity of hippocampal subfield volume cornu ammonis 2+ 3 over the course of withdrawal in patients with alcohol dependence. JAMA. 2014. Available:](http://paperpile.com/b/58tliY/Upoy) <https://jamanetwork.com/journals/jamapediatrics/fullarticle/1873180>

6.	[Frischknecht U, Hermann D, Tunc-Skarka N, Wang G-Y, Sack M, van Eijk J, et al. Negative Association Between MR-Spectroscopic Glutamate Markers and Gray Matter Volume After Alcohol Withdrawal in the Hippocampus: A Translational Study in Humans and Rats. Alcohol Clin Exp Res. 2017;41: 323–333.](http://paperpile.com/b/58tliY/ZkR1)

7.	[De Santis S, Bach P, Pérez-Cervera L, Cosa-Linan A, Weil G, Vollstädt-Klein S, et al. Microstructural White Matter Alterations in Men With Alcohol Use Disorder and Rats With Excessive Alcohol Consumption During Early Abstinence. JAMA Psychiatry. 2019;76: 749–758.](http://paperpile.com/b/58tliY/yEmp)

8.	[Pandey AK, Ardekani BA, Kamarajan C, Zhang J, Chorlian DB, Byrne KN-H, et al. Lower Prefrontal and Hippocampal Volume and Diffusion Tensor Imaging Differences Reflect Structural and Functional Abnormalities in Abstinent Individuals with Alcohol Use Disorder. Alcohol Clin Exp Res. 2018;42: 1883–1896.](http://paperpile.com/b/58tliY/jnpw)

9.	[Sameti M, Smith S, Patenaude B, Fein G. Subcortical volumes in long-term abstinent alcoholics: associations with psychiatric comorbidity. Alcohol Clin Exp Res. 2011;35: 1067–1080.](http://paperpile.com/b/58tliY/DsXu)
