# Supplementary material for: Hippocampal subfield volumes in abstinent men and women with a history of alcohol use disorder
Source: PLoS One. 2020 Aug 10;15(8):e0236641. doi: 10.1371/journal.pone.0236641 (PMC7416961; doi:10.1371/journal.pone.0236641)
Supplement: Attachment — Submitted filename: PONE-D-19-32308R2-reply.docx [file pone.0236641.s010.docx]

**Kayle S. Sawyer, Ph.D.**

**Boston University School of Medicine, L-1004**

**72 East Concord Street**

**Boston, MA 02118**

July 1, 2020

Stephen D. Ginsberg, Ph.D., Section Editor, PLOS ONE

Nathan S. Kline Institute for Psychiatric Research

New York, NY

Dear Dr. Ginsberg:

We thank the editor and the reviewers for their valuable comments regarding our manuscript entitled “Hippocampal subfield volumes in abstinent men and women with a history of alcohol use disorder” (PONE-D-19-32308R1). We appreciate the careful reading and attention to detail provided by the reviewers, and have implemented each of the four suggestions from Reviewer #2.

**Reviewer #2: The authors have done a good job of addressing most of my comments in the first review.**

Authors: Thank you for the careful reading. We agree with each point below and are glad you caught these details.

1. **Reviewer #2: We wish to note that Iglesias and colleagues had built the segmentation classifier used in the FS 6.0 subroutine with T1-only segmentation in mind, as described in their paper as follows:
   “The use of a simple, linear classifier such as LDA ensures that the classification accuracy is mainly determined by the quality of the input data (i.e., the subregion volumes) rather than stochastic variations in the classifier.”
   The line quoted from the paper does not imply in any way FS6 had only T1 segmentation in mind. In fact, contrary to that, the paper shows better performance when using both T2 and T1 and the whole extra contrast high in-plane resolution image as an additional input was developed to address the limitations of T1. Please remove this from Discussion or rewrite to reflect what Iglesias et al. have stated. "However, Iglesias and colleagues had built the 589 segmentation classifier used in the FS 6.0 subroutine with T1-only segmentation in mind (see 590 page 133 of their paper)."**

Authors: We agree that our writing was not clear. By stating that Iglesias et al. had T1-only segmentation “in mind,” we had intended to indicate simply that they had built the software so that it could function with T1-only datasets, not that this was their primary aim. We removed the sentence beginning “However…” and re-wrote the preceding sentence as follows: “The segmentation procedure we used (developed by Iglesias et al. [33]) generates better results when using both T2 and T1 than when using T1 alone, and our use of T1 scans without T2 scans could have resulted in lower accuracy.”

2. **Reviewer #2: "We found no gender-by-region-by-age interaction, nor any gender effects for individual subfields after correction for multiple comparisons. We have updated all the results in the text, and therefore, removed the gender specific results."
   Can the abstract be updated to incorporate this as one of the goals is to demonstrate gender specific effects which seem muted after the residual method? I also see no change in Discussion despite a lot of gender specific stuff taken out in the new version. Please also check Discussion to update based on the new results.**

Authors: In the Abstract, we added the following text: “Interactions with gender and age were of particular interest...,” and “...no significant interactions with gender were identified.” In the Discussion, we cut half the text from the paragraph regarding gender.

3. **Reviewer #2: The introduction is very rambling and can be easily shortened without losing its message. But more importantly, the final paragraph does not succinctly say what the authors set out to do. Instead it editorializes like "Ultimately, we hope that the findings will help lead to an increased understanding of how subregional hippocampal shrinkage contributes to altered memory abilities in AUD men and women as they age". This belongs in Discussion not in Intro. The first paragraph of methods (We examined.....) might make more sense at the end of the Introduction.**

Authors: We re-organized and condensed the Introduction as follows. The first paragraph (1) justifies the study. The subsequent two paragraphs (2 & 3) describe the subfield networks, to explain why we examined distinct subfields. Next, we summarize the AUD subfield literature (first authors Lee, Kühn, and Zahr), and include a condensed discussion of age and gender literature in this paragraph (4). We combined the investigation of memory and drinking history into one paragraph (5). Finally, we include a revised summary paragraph (6).

In that final paragraph, we deleted the editorializing sentence beginning with “Ultimately,” and moved the two sentences from the Methods to the summary of the Introduction: “We examined the interaction of group-by-gender-by-region to assess how the impact of AUD differed for men and women. Also, we examined the interaction of group-by-gender-by-age to assess abnormal aging for men as compared to women.”

4. **Reviewer #2: Statistical analyses- "Because brain volumes vary with head size, we used normalized volume values"-- You are not normalizing but rather correcting for ICV. Perhaps a carryover from the proportions method? Also please clarify that mean eTIV (and slope) was over the controls but the correction was applied to both groups just to be precise.**

Authors: We updated the text to read, “Because brain volumes vary with head size, we corrected for cranial volume, as follows: The NC volumes were fit to the estimated total intracranial volume (eTIV) values, and the slope ‘s’ was obtained from the NC group. Individual volumes for NC and AUD participants were adjusted using the formula V' = V − s × (eTIV − mean eTIV).”

**Reviewer #3: This revised manuscript examines volumes of hippocampal subfields in a sample of men and women with and without a history of alcohol use disorders. Analyses find several group differences in hippocampal subfield volumes, with main effects of group moderated by interactions with gender, age, and drinking phenotypes. I was Reviewer 3 on the original version of the manuscript. As for the original manuscript, the revised manuscript is very well written, the rationale is clearly laid out in the Introduction, relevant literature and background is reviewed, and the Discussion is well structured. The authors have been very responsive to my own comments/suggestions and to the other Reviewers. I particularly appreciate their transparency in posting data and code and revised analyses. The revised manuscript is a strong one with important implications for the field of alcohol research.**

Authors: We are grateful for the kind words.
